# Spontaneous Firings of Carnivorous Aquatic *Utricularia* Traps: Temporal Patterns and Mechanical Oscillations

**DOI:** 10.1371/journal.pone.0020205

**Published:** 2011-05-27

**Authors:** Olivier Vincent, Ivan Roditchev, Philippe Marmottant

**Affiliations:** CNRS/Université Grenoble 1, UMR 5588, Laboratoire Interdisciplinaire de Physique, Grenoble, France; University of Manchester, United Kingdom

## Abstract

Aquatic species of *Utricularia* are carnivorous plants living in environments poor in nutrients. Their trapping mechanism has fascinated generations of scientists and is still debated today. It was reported recently that *Utricularia* traps can fire spontaneously. We show here that these spontaneous firings follow an unexpected diversity of temporal patterns, from “metronomic” traps which fire at fixed time intervals to “random” patterns, displaying more scattered firing times. Some “bursting” traps even combine both aspects, with groups of fast regular firings separated by a variable amount of time. We propose a physical model to understand these very particular behaviors, showing that a trap of *Utricularia* accomplishes mechanical oscillations, based on continuous pumping and sudden opening of the trap door (buckling). We isolate the key parameters governing these oscillations and discuss the effect of their fluctuations.

## Introduction

Aquatic species from the genus *Utricularia* are widespread carnivorous plants, catching their preys with millimeter-sized traps. Since the discovery of their carnivorous character [1], [2], there has been much interest in the mechanism underlying their extremely fast motion: the entrance of a trap is closed by a door which is capable of opening and closing at the time scale of 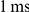 only [3]. It is known that slow pumping of water out of the trap enables storage of elastic energy in the trap walls, which is suddenly released when the trap is triggered by a slight touch on one of its four trigger hairs [4], [5]. However there is still debate on the mechanism at the origin of the door opening [6], [7]. Recent work has focused on time-resolved analysis of the door dynamics at small time scales, bringing to light the mechanical role of the door as a buckling valve[3], and long time analysis, showing that a single trap is able to fire spontaneously many times without any external action [3], [8]. In order to understand this surprising behavior and how it is connected to the trapping mechanism, we studied spontaneous firings of *Utricularia inflata* and *Utricularia australis*, recording the times of the firings and the temporal evolution of the trap shape. The aim of this paper is to present the original behavior of the recorded traps which proved to be much more complex than previously thought, and to show how these behaviors can be described by a simple physical model combining the deterministic mechanics of the elastic door and statistical fluctuations.

## Results

### Time repartition of spontaneous firings

**Figure 1:**
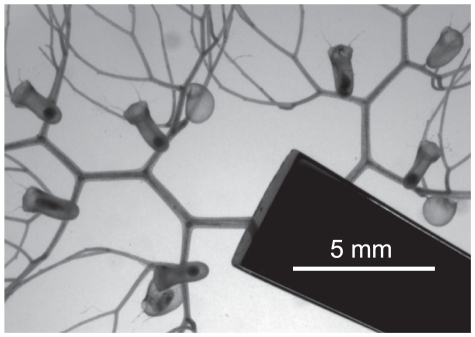
General view of an excised composed leaf of *Utricularia inflata*. The plant is held by tweezers (in black) and immersed in de-ionized water. See also Video S1.

**Figure 2:**
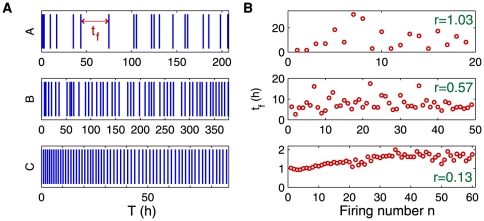
Extract of the firing events of 3 different traps of *Utricularia inflata*. Time (T) is set to 0 at the beginning of each sample. **A**: a vertical bar is drawn each time a firing occurs. **B**: corresponding time intervals 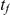 between successive firings. The value of the randomness index 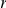 associated to each sample is indicated. For trap C, 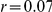 for the 20 first firings.

The plants were immersed in unstirred de-ionized water to avoid the presence of animals or fluid motion capable of triggering traps (see Figure 1 and Video S1). All observed traps showed spontaneous firings, with a maximum of about 200 firings for a single trap in three weeks. Some typical examples of their temporal behavior are presented on Figure 2. On Figure 2A, a vertical bar is plotted each time 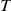 a firing occurs, for three different traps. Denoting 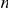 the firing number, we define the time interval between consecutive firings 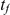 as 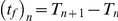. On Figure 2B, 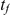 is plotted as a function of 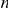. Both panels of Figure 2B show that different traps on a same composed leaf of *Utricularia inflata* can have very different behaviors. First, firings in trap A are spaced and scattered in time. This behavior will be called “random” in the following. On the contrary, “metronomic” traps such as trap C show very regular firings occurring at well-defined time intervals. The limit between these behaviors is sometimes thin, as shown by trap B: events are not well organized in time such as in trap C, but the time interval between firings is not as widespread as in trap A. This suggests that more than two distinct behaviors, “random” and “metronomic” traps are two extreme cases of a continuous range of behaviors.

We noticed that “metronomic” traps often show a slow drift of their period 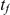, which is for example doubled after about 40 firings for trap C. This fact prevents to use the standard deviation of 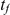 as an indicator of the behavior of a trap: much of the calculated standard deviation for trap C would indeed come from the regular drift of its period. To limit this bias, we define a “randomness index” as
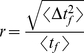
(1)where 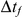 is the variation of time intervals for successive firings: 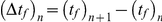 and 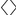 represents an average over all successive firings of the considered sample. Values of 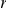 for traps A, B, C are shown on the right panel of Figure 2, showing that the visual feeling of randomness is well reproduced by the value of 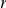, which is less than 0.1 for very “metronomic” traps and of the order of 1 for very random traps.

**Figure 3:**
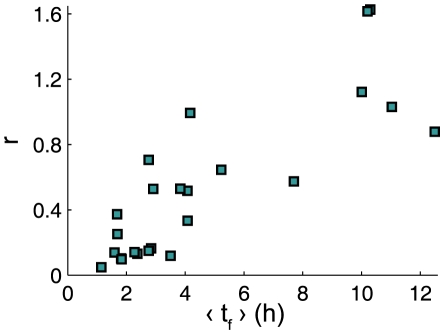
Randomness index 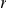 versus mean firing time interval 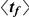. Values of 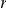 and 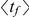 are calculated for 24 samples containing between 10 and 107 firings from 20 different traps of *Utricularia inflata*.

Noticeably, the last presented firings of trap C become more scattered as 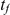 increases, as was also observed on other “metronomic” traps. This suggests a link between the irregularity of a trap, characterized by 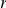, and its period 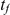. To check this hypothesis, we calculated 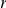 and the mean value of firing intervals 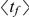 for several samples from 21 different traps of *Utricularia inflata*. Results are presented on Figure 3 and confirm the tendency of higher irregularity for higher firing periods. “Metronomic” traps (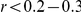) typically fire between 1 h and 3 h on average, while “random” traps (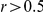) display values of 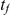 usually bigger than 5 h.

**Figure 4:**
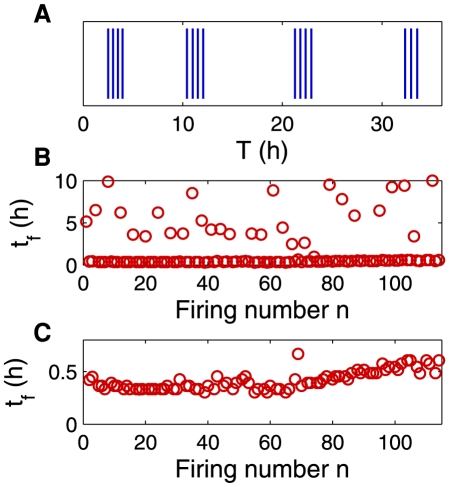
“Bursting” behavior in trap D (*Utricularia australis*). **A**: excerpt of the firing events. A vertical bar is drawn each time a firing occurs (time is set to 0 at the beginning of the sample). Firings occur by bursts of 3 or 4 events. **B**: time intervals 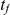 between successive firings for all the recorded firings. The scattered points correspond to the times between consecutive bursts, while the regular line at the bottom is drawn the very regular firings inside a burst. **C**: Magnification of the bottom line of panel B. The associated randomness index is 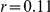.

Looking closer at some apparently very fluctuating traps, we found that some of them displayed a surprising grouping effect, where firings often happen by groups of 2 up to 7 very close and regular events, separated by a variable amount of time. In our experiment with *Utricularia inflata*, five traps presented these “bursts”, but the most striking example was given by a trap of *Utricularia australis* (trap D) followed ten consecutive days (see Figure 4A and Video S2). The time intervals between firings 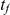 measured on this experiment are shown on Figures 4B and 4C, showing that time intervals between consecutive events inside a burst follow a very regular line as for “metronomic” traps, with a randomness index 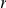 close to 0.1. On the contrary, the time separation between bursts is very scattered as for “random” traps. Note that the number of firings per burst is roughly constant, 3 or 4 in this case, and that 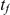 inside a burst is very small, of order 30 min. Interestingly, even for *Utricularia inflata* traps, this latter time is small, typically between 15 min and 1 h. This suggests that bursts might be a characteristic behavior for high-frequency (low 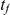) traps.

During the three weeks of observation, traps have not shown significant changes of behavior. Slow transitions from “metronomic” to more “random” periods often happen, correlated with an increase in the time between firings 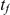. Also, a few traps stopped firing after a few days, sometimes temporarily, sometimes definitely. We will refer to these latter traps as “waiting” in the following.

In conclusion, our experiments exhibit a rich variety of behaviors, the most surprising ones being “metronomic” spontaneous firings, following precise temporal patterns, and “bursting” ones which combine regularity and randomness at different time scales. We suggested above that the mean time interval between firings was an important parameter determining the behavior of a trap.

### Study of the change of width of the traps

**Figure 5:**
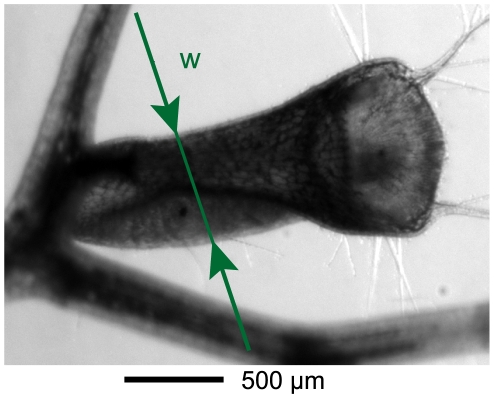
View of trap B (*Utricularia inflata*). The figure shows the definition of the lateral width 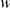 used for the data presented on Figure 6.

**Figure 6:**
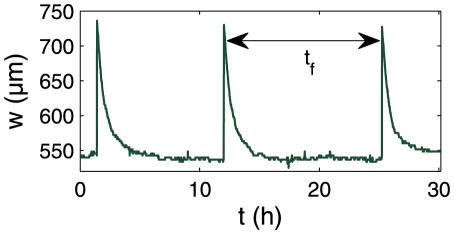
Extract of the evolution in time of the lateral width 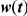 of trap B. Three successive spontaneous firings can be observed.

**Figure 7:**
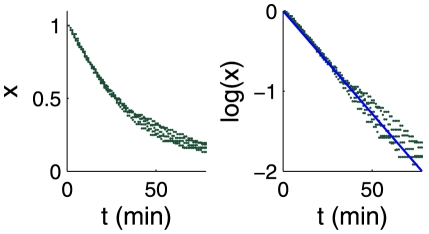
Degree of inflation 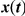 for 5 successive firings of trap B. 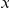
 is defined as 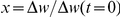 where 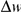 is the difference between the current value of the trap lateral width 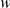 and its value at full deflation. Left: linear plot. Right: logarithmic plot. The blue line corresponds to a fit of 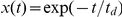 with 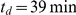. Time t is reset to 0 at each firing.

**Figure 8:**
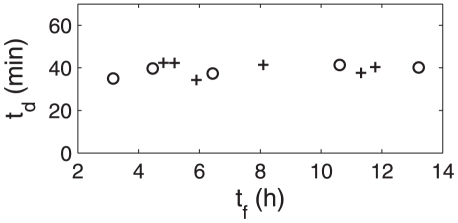
Measured deflation time 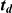 for 12 different firings of trap B versus the corresponding firing time interval 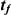. Two series of 5 and 7 firings are shown (first series in circles, second series in crosses), and are separated by five days. Uncertainties on 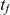 are negligible compared to those on 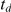 (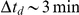).

In order to understand more in detail the origin of the behaviors described above we focused on the physical process of trap setting: due to active pumping of water, the volume of the trap decreases with time, thereby lowering the pressure inside the trap. We thus extracted when possible a measurement of the width of the traps as a function of time 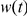, obtained by image analysis on traps viewed from above (see Figure 5), so it represents a projected width, used as an indicator of the trap state. The curve obtained for trap B is shown on Figure 6. Each peak corresponds to a spontaneous firing, followed by a decrease of the trap width from its inflated state to its deflated state. As shown in a previous paper [9], this relaxation is exponential, with a characteristic time 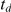. This is well verified in our experiments, as can be seen on Figure 7. We also checked that 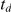 did not vary much for a single trap, and that its variations were not related to those of the time intervals between firings (see Figure 8). This shows that the source of fluctuations in 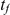 has to be found elsewhere, and that 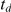 can be considered as a constant, characteristic deflation time for the considered trap.

Similar results were found on other traps of *Utricularia inflata* and *Utricularia autralis*, and some values of 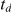 are reported in table 1. Since 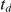 is a natural unit of time for each trap, it is interesting to measure the firing intervals 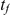 in units of 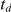: the ratio 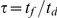 indicates at which level of the deflation process firings occur. Our results for “metronomic” and “random” traps presented in table 1 show that 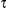 seems to be strongly linked to the behavior of a trap : the higher the value of 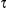, the higher the irregularity of the trap. Observations on other traps where 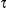 was not precisely measurable show this general trend: “metronomic” traps fire at an early stage of the deflation process and “random” traps usually fire close to their fully deflated state.

**Table 1:** Deflation times and firing intervals.

trap	A	B	C′	D	
type	random ( 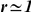 )	random ( 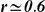 )	metronomic ( 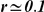 )	burst (intra)	burst (inter)
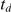 (h)	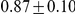	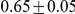	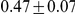	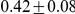	
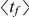 (h)	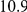	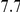	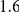	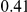	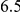
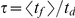	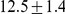	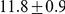	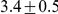	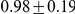	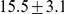

Typical values of deflation times 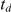 and the mean value of firing intervals 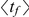, calculated over successive firings of the considered trap. Due to the angle of observation, 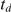 was not precisely measurable on trap C so another “metronomic” trap was considered, denoted C′. For trap D, we distinguish the firing time inside a burst (intra) and between bursts (inter).

**Figure 9:**
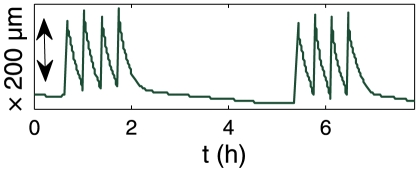
Extract of the evolution in time of the lateral width of trap D (*Utricularia australis*). Two successive bursts comprising 4 firings each are shown. See also Video S2.

We now focus on “bursting” traps such as trap D. The evolution of trap thickness presented on Figure 9 displays the same characteristics: inside a burst, successive firings are fast and complete deflation is never achieved (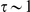), visible in the fact that the slope of the curve of Figure 9 is always considerable, whereas the time interval between two bursts is long compared to 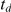 (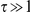), as can be seen in the last two rows of table 1. We noticed that *Utricularia inflata* bursting traps also feature 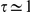.

We thus suggest that instead of simply 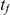 (see previous section), the relevant parameter predicting the behavior of a trap is 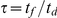: high values of 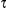 correspond to an irregular behavior, while low values are associated with regular traps. In the following discussion, we develop physical arguments supporting this hypothesis.

## Discussion

Our experiments show a very rich variety of behaviors in traps of *Utricularia inflata* and *Utricularia australis*. Environmental fluctuations such as day/night oscillations, temperature changes or light intensity variations cannot account for these observations, since all observed traps were on a same composed leaf under the same conditions. To explain our observations we have to understand how the trapping mechanism works. It has been put forward that the opening of the trap door of *Utricularia* was based on an elastic instability: buckling, which is a mechanical process where an elastic membrane, in our case the trap door, resisting a pressure difference 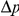 suddenly changes its curvature at a critical pressure difference 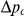 [3]. We will explain our experimental results in the light of these considerations, suggesting that the repetitive character of observed firings is a direct consequence of the spontaneous buckling of the trap door.

### Buckling cycles: “metronomic” and “waiting” traps

**Figure 10:**
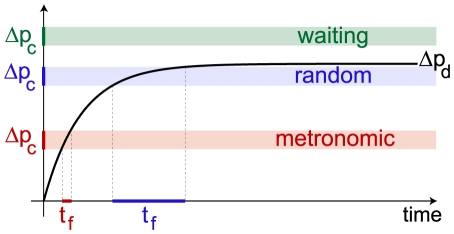
Model explaining the trap behaviors. The black curve is the evolution of the pressure difference 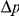 due to the deflation process, saturating at a value 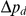. Firing of the trap door occurs at a time 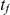 when 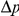 reaches the buckling pressure 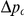. Fluctuations in 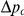 entail fluctuations in 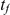 which are bigger when 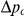 is close to 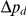, explaining the scattered values of 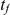 for “random” traps. If 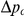 is bigger than 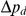, buckling is impossible and the trap is in a “waiting” state.

Our results confirm previous observations [3], [8], [10] that deflation starts immediately after firing. Since deflation originates from active pumping of water out of the trap [5], this indicates that pumping is a continuous process. Starting from a fully inflated trap, this continuous pumping entails a progressive deflation, represented by 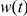. As a consequence, the pressure inside the trap lowers to a pressure 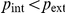, where 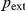 is the pressure in the surrounding liquid. This entails an increase of 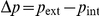, representing the net pressure exerted on the trap door. If there is a well-defined pressure difference 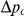 at which the trap door buckles, then the door spontaneously opens when 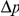 reaches that value. The door being open, the trap inflates and the pressure difference is reset to zero. As the door closes, the same cycle of deflation - buckling can start again, *ad infinitum* This picture shows how the observed “metronomic” oscillations of the trap width may arise from the combination between continuous pumping and spontaneous buckling of the trap door. Note that if 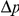 is never high enough to reach 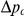, then the trap never fires spontaneously and stays in a “waiting” phase (see Figure 10). Experimentally, the hypothesis of door buckling is supported in our experiments by the fact that the level of deflation achieved when a firing happens does not vary much for successive firings of a single trap (see Figure 9 for example), meaning that firings probably occur at comparable 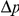. We now derive a simple model to extract the physical parameters governing these oscillations.

First, we have to understand the evolution of the pressure difference 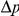 in time. Our experiments only access to the trap width 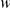, but due to the elasticity of the trap wall, 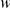 is directly linked to 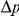 with a relationship that we assume linear (see Methods). Consequently, since 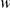 decays exponentially with a time constant 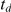, one should also have
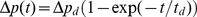
(2)

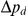
 being the maximum pressure difference attainable by the trap, corresponding to a fully deflated state. The value of 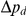 has been estimated to 16 kPa [10].

Note that expression (2) can be predicted theoretically using simple hypotheses (see Methods). The model presented in the Methods section also justifies our experimental observation that 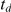 does not vary much in time for a single trap, since it does not depend on the pumping rate but mainly on the permeability and elasticity of the trap body, which can be considered as constant.

The spontaneous firing of the trap occurs at a time 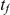 where 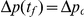, which is possible only if 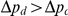, i.e. when pumping is strong enough to make the door buckle. If this is the case, then from equation (2) we have
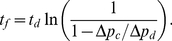
(3)


As we demonstrated in our experiments, 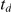 is constant for a trap, so that 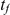 only depends on the ratio 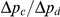. If 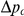 and 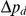 are constant, 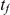 is a constant too and firings occur at very regular intervals: this corresponds to the “metronomic” behavior. Notice that this metronomic characteristic doesn't depend on the mathematical expression of 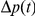, and is always true if the evolution of 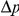 in time is the same after each firing, which is justified by our observations that 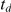 is a trap constant, and if there exists a time 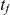 where 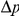 reaches the critical buckling pressure 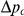. If this latter condition is not verified, the trap is not able to fire spontaneously and is in a “waiting” state.

### Fluctuations and “random” traps

In order to explain random firings with this model it is necessary to introduce fluctuations. At these scales, buckling is insensitive to thermal noise [9] and for an incompressible spherical shell with a thickness 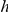, a radius 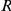 and a Young's modulus 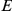, the critical buckling pressure is given by [11]

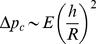
(4)so any change in elasticity, affecting 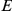, or shape, affecting 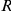 but also probably the exact prefactor in equation (4), is able to impact the value of 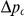.

Changes in shape can occur at each firing since the trap door doesn't necessarily come back exactly at the same position when it closes. Changes in elasticity are also possible if there are variations of turgor pressure inside the door wall. It is clear from equation (3) that fluctuations in 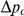 directly impact the time interval between firings 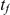. Figure 10 shows how fluctuations in 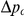 affect the distribution of 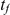. In particular, fluctuations around a small value of 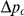 have a much weaker effect than the same fluctuations around a value of 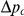 close to 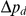, due to the exponential evolution of 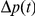. Thus “metronomic” traps should have a low value of 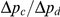 or equivalently a low value of 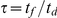 (see equation (3)), while “random” traps would have 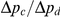 closer to 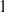, leading to a higher value of 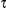, which is well supported by our experimental results.

This model also predicts that if the mean firing period of a single trap increases, fluctuations of the firing times should also increase. Trap C provides a good illustration of that point on Figure 2B, bottom. It could also be an explanation of our observation that as time passes, “metronomic” traps often slow down their firings, leading them to become more “random”, temporarily or permanently.

**Figure 11:**
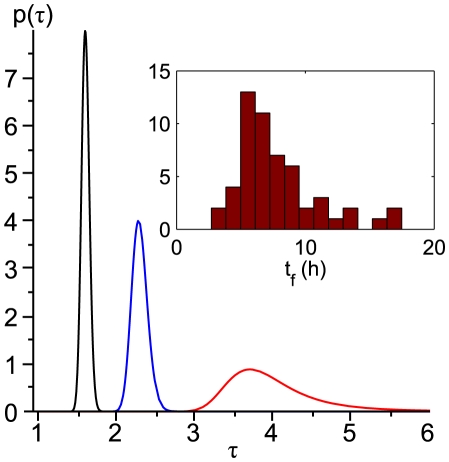
Probability distributions on 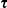. They are calculated assuming gaussian fluctuations on 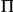 with standard deviation 0.01 and centered on 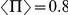 (black), 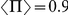 (blue), 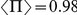 (red). The distribution on 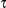 becomes broader as 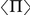 increases, but also less symmetrical, as observed for “random” traps: a histogram of the firing times 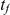 of trap B is presented in the inset of the figure for qualitative comparison.

Notice that 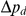 could also fluctuate on the same order of magnitude than 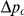, due to changes in the pumping rate for example. However, one cannot actually separate the effect of 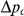 and 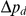 as can be seen from equation (3), and the relevant parameter is in fact 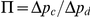. Using the other natural parameter 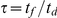, equation (3) reduces as
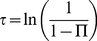
(5)and we now derive some properties of such a dependence between 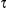 and 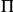, keeping in mind that the detailed results depend on the exact expression of 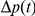. However this simple expression helps us to illustrate the previous arguments. Moreover, the ideas developed below remain valid for any 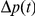 provided that 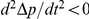. For example one can calculate how fluctuations propagate from 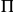 to 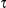 (see Methods), and one can readily show, assuming 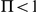, that the mean values 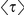 and 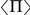 follow equation (5), and that the standard deviations 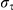 and 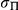 of respectively 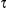 and 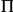, are related by
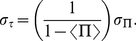
(6)Due to the denominator 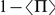, fluctuations get largely amplified as the mean value of the pressure ratio 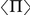 gets closer to 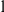. Within the framework of this model, the wide range of time intervals between firings of some traps (the “random” ones) just reflects the amplification of pressure fluctuations which become very important when the buckling and pumping pressure 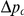 and 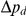 are comparable. We also show that in addition to the amplification of fluctuations, the shape of the probability distribution is modified (see Figure 11). In particular, symmetrical distributions on 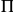 give distributions on 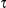 that expand towards large values of 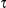, explaining the non-symmetrical aspect of the repartition of firing times 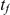 in random traps (see inset of Figure 11).

Fluctuations around 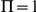 can also occur, making the trap oscillate between “waiting” and “random”, giving much more scattered events, which is probably the case for trap A which displays long waiting periods and a large value of 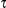.

Interestingly, even “metronomic” traps have 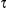 bigger than 1, meaning that 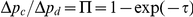 is never far from 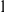. Thus, all traps seem to have 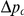 comparable to 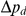. The reason could be that a too low 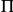 would have the trap firing very often but not achieving significant deflation: only a small amount of liquid would be sucked at each firing making the trap not efficient to catch preys. On the other hand, a high value of 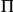 would mean that the deflation pressure 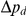 is small compared to the buckling pressure 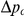, making the door wall very stable and the trap difficult to trigger. This completes the discussion presented in [3] showing how the elasticities and shapes of the trap wall and the door are optimized for efficient prey capture.

### Bursts

At first sight, bursts could also be interpreted as fluctuations of time intervals. However, the facts that time intervals in the bursts are very well defined and that the number of peaks in a burst is roughly constant rules this idea out. In a burst, the behavior of a trap strongly looks like a “metronomic” one. Looking at a larger time scale, bursts groups seem on the contrary to be randomly distributed. To account for this unexpected behavior, we suggest that after a trap is triggered, there should be a process relaxing with a time 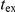. For example, 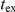 could be a typical relaxation time of the door rigidity *via* its turgor pressure. Thus, any firing of the trap would be associated with a reduction of the buckling threshold 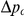 for a time 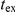. When triggered or spontaneously fired, such a trap would go from “waiting” or “random” to a “metronomic” state for a time 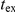 due to the lowering of 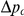, thus 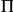, then go back to its initial state, giving the observed bursts. This hypothesis is supported by the experimental fact that the number 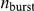 of firings per burst is usually constant over long periods of time: this number would be approximately given by 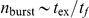.

**Figure 12:**
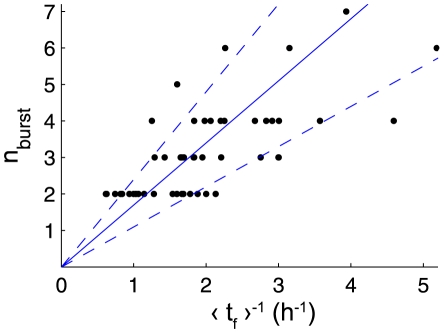
Number of firings inside a burst versus firing frequency. The firing frequency is defined as the inverse of the mean time interval between firings inside a burst 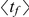. It is calculated for 53 bursts of 5 different traps of *Utricularia inflata* and one trap of *Utricularia australis*, along with the number of firings per burst 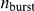. Blue lines correspond to 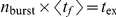 with values of 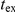 of 1.7 h (central line), 1.1 h (lowest dashed line) and 2.4 h (upper dashed line).

To check this, we analyzed 53 bursts on six different traps, and recorded 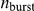 and the mean value of firing intervals 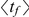 inside the considered burst. We then plotted 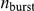 versus 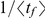 (see Figure 12), the slope of which should be approximately 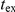. We find a good agreement with this prediction for a wide range of 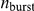, comprised between 2 and 7, and we deduce that 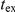 should be of order 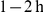. The scattering of the points on the plot probably comes from the variation of 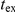 from trap to trap, but also for a single trap in time.

Note that if the process related to 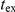 was triggered at each firing, the started burst would never end: it is here implicitly assumed that the excitation process cannot be reactivated until it has fully relaxed.

When 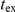 is smaller than 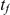 or of the same order, there is only one firing per burst, which means that no burst is observed. This should be the case for usual “random” or “metronomic” traps which present no bursts. Indeed, as seen on Figure 3, the fastest non-bursting traps have 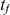 of order 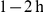 which is also the value of 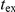 calculated above.

Bursts in *Utricularia* seem to be an evidence of a sensitive process occurring during firings of its traps, suggesting that in addition to 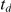 there is another characteristic time of a trap 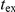 to take into account. We hypothesize that bursts are likely to happen when the parameter 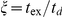 is greater than 1, since spontaneous firings happen with a time interval of order 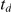 or more. However, there are still open questions to explore, either on the precise chemico-physical mechanism explaining the origin of 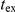 or on the benefit bursts could bring to the plant: is it a way to increase the capture rate of a trap when animals are close, or is it just an unavoidable effect of the global trapping mechanism of *Utricularia*?

### Conclusion

Early investigations on spontaneous firings of *Utricularia* traps suggested that they were randomly distributed in time. We proved here that these apparently random distributions were just one aspect of a larger set of behaviors, which can be very regular and organized in time. All these behaviors can be found on different traps of a composed leaf, suggesting complementary roles: in addition to catching occasional preys as “waiting” and very “random” traps do, the very regular firings of “metronomic” traps could be a way to diversify the plant's alimentation by continuously catching smaller organisms not capable of triggering the trap by themselves, such as phytoplankton or bacteria. This underlines the importance of these organisms for the plant's nutrients supply, as recently suggested [12], [13].

We also proposed a physical model, showing how the short and long time behavior of the traps were connected: fast opening of the door and spontaneous firings are just two consequences of a single aspect which is the buckling of the door wall. Thus, to achieve its regular firings without any active signal or feedback, the plant simply uses mechanical oscillations, which only ingredients are continuous pumping and buckling of the trap door. Based on this idea, the different trap types can be explained by introducing fluctuations in the mechanical parameters, which occur naturally due to biological or geometrical changes. The key parameters to predict the behavior of a trap are 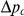, the critical pressure at which its door buckles, and 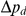, the maximum pressure difference it can generate by active pumping. We suggested that 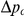 and 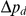 were always of the same order, optimizing the trap efficiency, and that their relative values condition the temporal aspect of firings. It has to be noted that even if our model strongly supports the idea of buckling as the mechanism for firing *Utricularia* traps, it doesn't exclude any sensitive effect of the trigger hairs, which could act chemically or mechanically to facilitate buckling. Note that the presence of a sensitive process is also suggested by the bursting behavior of some traps.

This ingenious way to create a periodic signal, recalling some aspects of Tantalus vase, could provide biomimetical inspiration for autonomous elastic structures, and represents in itself an original illustration of mechanical oscillators for an undergraduate Physics course.

Hopefully this work will stimulate further collaboration between biologists and physicists to clarify completely the mechanical and biological processes at the root of the unique trapping mechanism of *Utricularia*. One big challenge is a direct, non destructive measurement of the pressure inside the trap, which is for now only accessible by looking at the trap width. Future work could also be directed towards the characterization of the bio-chemical response resulting of action on the trigger hairs, or of temporal behaviors for other *Utricularia* species.

## Materials and Methods

### Preparation of excised leaves

Composed leaves from aquarium-cultivated *Utricularia inflata* and *Utricularia australis* obtained from “Nature et Paysages”, France, were excised keeping bladders, and carefully washed with deionized (DI) water before immersion in a Petri dish filled with DI water. Special care was taken to avoid accidentally triggering the traps when transferred, usually leading to the aspiration of an air bubble. The leaf was held at the bottom of the petri dish by inoxidable tweezers. Volumes of DI water used in the experiments were small (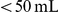), so we cannot exclude the presence of solutes such as minerals in an unknown concentration, brought by the plant itself for example. As a matter of fact, authors of previous studies of *Utricularia* cited in this article (see for example [8], [10]) usually add a small quantity of ions in water to reproduce natural living conditions. However, since the excised leaves continued to live and grow for more than three weeks and most of the traps presented regular deflation - firing cycles, our liquid medium was probably adapted, even if not optimized.

### Observation

The Petri dish rested on a LED Backlight device (from LEICA, France) consisting of 20 6-watts white LED at color temperature 5000K, distributed on a 55 mm disk under a light diffuser. Such constant illumination was used to avoid any effect due to ambient light. Images were recorded with a time-lapse camera, allowing observation of *Utricularia* traps for long times, of the order of several weeks. Petri dishes were not covered to avoid condensation, so they had to be regularly refilled with care, typically each week. The room temperature during observation was 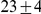 degrees Celsius and no effect of temperature variations on the trap behaviors was observed.

Two composed leaves of *Utricularia inflata* respectively containing 10 and 12 traps were followed continuously during 3 weeks. All of the 22 observed traps showed spontaneous firings, even if 6 of them stopped firing after 1 to 3 days. Among these 6 latter traps, 2 traps fired again a few days later, showing that they were still working. One trap also oscillated between periods of firings and periods of apparent inactivity, each during about 3 days. All other traps had constant firing activity.

Two other experiments were conducted with single traps: one with *Utricularia inflata* (trap B) and one with *Utricularia australis* (trap D).

On these 24 traps, a total amount of 1549 spontaneous firings were recorded. The bursting behavior was observed on 6 different traps.

### Image analysis and data processing

Image and data were processed using ImageJ freeware and Matlab (Mathworks), to extract the times at which observed firings occur, the time intervals between firings and their distribution.

If possible, the evolution of the trap thickness in time was also recorded, by extraction of the lateral dimension of a thresholded image of the trap. The characteristic pumping time 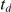 was then calculated by exponential fitting on these curves: if we define the deflated state 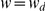 as the value of the exponential plateau (since that due to spontaneous firings, 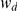 is never attained, its value has to be manually adjusted) and if 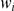 is the inflated width, we define a degree of inflation
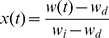
(7)which value is 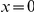 for a fully deflated state, and 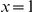 for an inflated state, just after any firing. 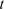 is reset to zero for each firing so that one has
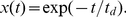
(8)Uncertainties on 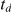 represent the standard deviation of the fitting parameter estimated in the regression process. Figure 7 presents on a same graph five successive firings of trap B showing that the deflation process is identical after all firings.

Some values of 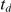 were also determined graphically with the methods of tangents, uncertainty is then an estimate of the error made on the slope of the curve at its origin. For the purpose of this article, precise determination of values and their uncertainties is not essential and the order of magnitudes extracted are enough to discuss the results.

For purposes involving more precise measurement of the trap width, the image analysis technique could also be used. Its precision is relatively poor for small magnifications (Figure 1 for example), due to the important pixel size: in this case, the precision of the measurement of 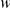 is of the order of 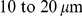, but it can be greatly improved using higher magnifications. The measurement of 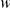 as shown on Figures 5 and 6 has a precision of 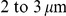. The drawback of using high magnifications is the loss of the ability to follow several traps at the same time.

Compared to the linear position sensor used in [8], the image analysis technique has the advantage to avoid any direct contact with the plant, but it only accesses a projected width of the trap, making it sensitive to any natural rotation of the trap. The combined use of these two techniques should thus be advantageous.

### Trap pumping and pressure evolution

The observed saturation of deflation to the fully deflated state shows that there is a process balancing pumping for high deflation. Two hypotheses can be formulated: either the pumping rate depends on the pressure difference 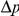 between the inside and the outside of the trap, so that pumping could be significantly lowered in the deflated state, or there is an incoming water flow balancing pumping due to porous fluid transport. Since lateral walls of the trap are thin, the latter is probable. We show that these processes can explain an exponential decrease of pressure inside the trap, using simple hypotheses: assuming that water is expelled from a trap with a constant flow rate 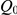, the volume 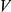 of water inside the trap should decrease linearly with time as 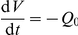. However, if the wall of the trap is porous, there will be an incoming flow rate 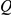. Due to the slowness of the pumping process and the small lengthscales involved, Darcy's law should be verified, and 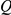 should directly be proportional to the pressure gap 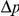:
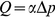
(9)with
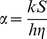
(10)where 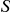 is the surface of the trap, 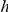 and 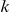 are respectively the thickness and permeability of the wall and 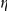 the viscosity of water. The volume conservation equation thus implies
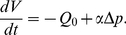
(11)


We now assume that due to the elasticity of the trap wall, there is a linear relationship between pressure and volume such that 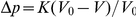 where 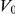 is the initial inflated volume of the trap, and 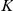 is a positive effective elasticity modulus (in Pa). This hypothesis was justified by numerical simulations with realistic *Utricularia* shapes [3], showing that 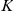 was constant except for very small deflations (volume change inferior to 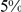). These simulations also showed that the trap volume 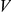 and width 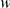 are proportional, which is due to the fact that the trap deforms mainly in the lateral direction (indicated by the arrows on Figure 5). As a result, the assumption of linearity between 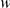 and 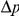 used in the Discussion section is reasonable.

Equation (11) then rewrites as
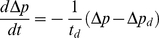
(12)with
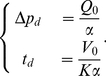
(13)


We recognize here a first order differential equation which admits (2) as a unique solution for the initial condition 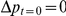.

From equation (13) we can estimate the trap permeability 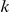 using equation (10) rewritten as 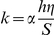 and using typical values for an *Utricularia inflata* trap: 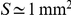, 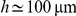, 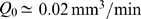, 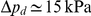 and the viscosity of water 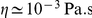, one finds 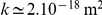 (see [9] for a similar estimation). If trap permeability is not the only phenomenon causing leaks, the flow rate 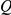 due to porous leaks should be lower, so that this estimate is a maximum value for the trap permeability. Notice also that due to the inhomogeneous character of the trap wall, the obtained value is an equivalent permeability averaged over all its surface and thickness.

This model is consistent with the experimental value of 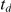 : using 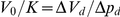, 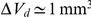 being the difference of volume between the inflated and deflated state, and equation (13), one finds 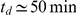.

At last, note that the exponential evolution of pressure is also compatible with a model (not detailed here) using the hypotheses of zero permeability of the trap wall and of a pumping rate 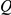 depending linearly on 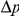.

### Permeability and equivalent radius

In the above paragraph, we showed that a trap could not go beyond a maximum pumping pressure 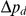 due to porous leaks. However, leaks could also come from a single hole of radius 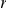 in a perfectly impermeable trap. Then if the Reynolds number (see below) is sufficiently low, 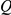 is also proportional to 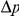 creating a Poiseuille flow with hydraulic resistance
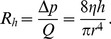
(14)Since 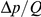 was equal to 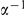 in the permeability model above, we find using equations (10) and (14) that
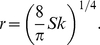
(15)Using the previous value of 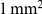 for 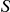, one finds 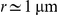.

The Reynolds number is expressed by 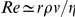 where 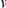 is the fluid velocity in the hole and 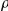 the fluid density. If there is a flow rate 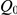 through the hole of raduis 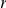 we should have 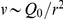 so that we have 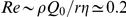. The approximation of Poiseuille flow is thus justified.

Notice that the values of the permeability 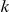 is very low, meaning that the water fluxes in and out of the trap are very small. As can be seen on the equivalent hydraulic radius of 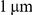, the trap door has to be perfectly closed to avoid any opening of this order of magnitude. This also underlines the difficulty of intrusive measurements of the inside pressure of the trap such as those of [10] which are likely to give biased results, since any hole of order 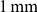 entails water fluxes comparable to the maximum ones that naturally occur. Such a provoked leak would considerably lower the observed value of 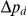.

### Fluctuation propagation between 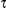 and 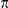


We assume here that probability distributions are smooth enough so that mean values and standard deviations are defined.

Expanding 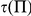 around 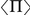 at the second order in 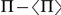 and taking the average one finds
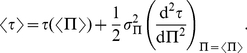
(16)Notice that the first order term is cancelled in the averaging process. Using the expression of 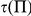 given in equation (5), one finds that the second order term is negligible when
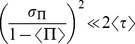
(17)so that the mean value of 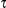 is simply given by
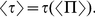
(18)


This allows calculating the mean value of 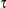 knowing the mean value of 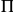, or by reversing the equation deducing the mean value of 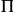 by measuring the mean value of 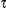 experimentally.

Standard deviation can also be calculated with the same Taylor expansion technique. The result for 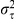 brings into play a sum of terms proportional to 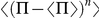, starting at 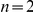. The 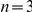 term equals 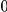 for symmetrical distributions of 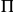, which we will assume in the following. This is also the case for all odd terms. Even terms are of order 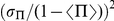 with respect to the previous even term so that one can keep only the term 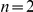 at the condition
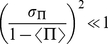
(19)giving the simple result
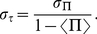
(20)Notice that conditions (17) and (19) can be rewritten respectively as 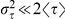 and 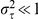. In our experiments 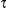 is always bigger than 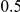 so that the second condition is the most restrictive.

Comparison with experimental values is difficult for several reasons: first, the previous inequalities are not verified for “random” traps. “Metronomic” traps have smaller fluctuations but these are difficult to measure, since it is not easy to separate actual fluctuations from the natural drift of the firing period. Second, results strongly depend on the exact mathematical relation between 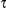 and 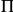, which is not accessible experimentally for now, especially for long times.

### Probability distributions

Assuming that the variable 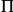 has gaussian fluctuations with standard deviation 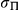, the associated probability distribution is
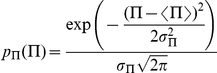
and one has the relation 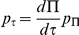 which gives, using equation (5):
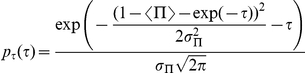
which is a function of 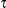 and of the parameters of the initial 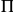 distribution: its mean 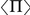 and its standard deviation 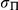. On Figure 11, 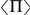 and 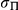 are chosen to illustrate the basic properties of such a distribution, namely the amplification of its standard deviation as 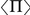 increases, and its asymmetry.

## Supporting Information

Video S1
**Spontaneous firings of** ***Utricularia inflata*** **traps.** This is the animated version of Figure 1. Ten traps of a same branch of *Utricularia inflata* were immersed in de-ionized water and their spontaneous firings were recorded with a time-lapse camera. The field is about 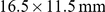 and the video is accelerated 1680 times (real duration: 11 hours and 12 minutes).(AVI)

Video S2 **Bursts in trap D (** ***Utricularia autralis*** **).** This video corresponds to Figure 9. This trap of *Utricularia australis* was recorded with a time-lapse camera and presented regular bursts of 3 or 4 spontaneous firings. Two bursts of 4 firings are present in the video. The trap is approximately 1 mm long and the video is accelerated 1680 times (real duration: 7 hours and 47 minutes).
(AVI)
